# Post‐TBI cognitive trajectories: Differences by sex/gender, cause, and severity

**DOI:** 10.1002/alz70860_107614

**Published:** 2025-12-23

**Authors:** Douglas William Hanes, Sean A.P. Clouston

**Affiliations:** ^1^ Renaissance School of Medicine at Stony Brook University, Stony Brook, NY, USA; ^2^ Dalla Lana School of Public Health, University of Toronto, Toronto, ON, Canada; ^3^ Stony Brook University, Stony Brook, NY, USA

## Abstract

**Background:**

While traumatic brain injury (TBI) is recognized as an important risk factor for cognitive health, most research has been conducted on military servicemembers and athletes, often postmortem—primarily (or exclusively) male populations with limited causes of TBI, access to healthcare and monitoring not typical of the general population. The generalizability of these studies, and the impact on TBI at the population level, remains unclear; and little research has been conducted on TBIs among the general population, including women, nor using non‐postmortem methods.

**Method:**

Using TBI and cognition data from the Health and Retirement Study, this paper analyses longitudinal changes in cognition and dementia risk among participants who have experienced TBI after age 50, including differences by sex/gender, TBI cause, and severity. Cognition was measured using a 27‐point scale and included immediate and delayed recall, numeracy, and orientation questions. For TBI, participants were asked a series of questions about whether they had had a traumatic brain injury, the date of the injury, cause of that injury, and various sequelae. A dummy variable was coded into having had a TBI or not, and variables were created to indicate the earliest and latest TBI each participant had. Sex/gender was gather in the initial wave and two options (male and female) were given.

Multilevel regression was conducted with cognition as the outcome variable and including time since TBI, source of TBI, and sequelae count, as well as sex/gender. Demographic variables, including age, race/ethnicity, education, wealth, veteran status, and histories of homelessness and incarceration were also included.

**Result:**

In the fully adjusted model, years‐since‐TBI was statistically significantly associated with a decrease in cognition (b = ‐0.05, *p* = 0.008). Interpersonal violence as the cause of TBI was also negatively associated with cognition (b = ‐0.74, *p* = 0.004). Neither sex/gender nor TBI sequelae had a statistically significant association with cognition

**Conclusion:**

TBI may have long‐lasting effects on cognitive health well after its occurrence, and these may be compounded when that injury is due to interpersonal violence. More research is needed to analyze the relationship between violence, TBI, and later‐life cognition.

